# Trophic interactions induce spatial self-organization of microbial consortia on rough surfaces

**DOI:** 10.1038/srep06757

**Published:** 2014-10-24

**Authors:** Gang Wang, Dani Or

**Affiliations:** 1 ETH Zurich, Institute of Terrestrial Ecosystems, Universitaetstrasse 16, 8092 Zurich, Switzerland

## Abstract

The spatial context of microbial interactions common in natural systems is largely absent in traditional pure culture-based microbiology. The understanding of how interdependent microbial communities assemble and coexist in limited spatial domains remains sketchy. A mechanistic model of cell-level interactions among multispecies microbial populations grown on hydrated rough surfaces facilitated systematic evaluation of how trophic dependencies shape spatial self-organization of microbial consortia in complex diffusion fields. The emerging patterns were persistent irrespective of initial conditions and resilient to spatial and temporal perturbations. Surprisingly, the hydration conditions conducive for self-assembly are extremely narrow and last only while microbial cells remain motile within thin aqueous films. The resulting self-organized microbial consortia patterns could represent optimal ecological templates for the architecture that underlie sessile microbial colonies on natural surfaces. Understanding microbial spatial self-organization offers new insights into mechanisms that sustain small-scale soil microbial diversity; and may guide the engineering of functional artificial microbial consortia.

Soil microorganisms are constrained to life in a fragmented and highly dynamic aqueous network in complex pore spaces that limit nutrient diffusion pathways, control cell dispersion rates, and shape interactions among microbial populations^1^,^2^,^3^,^4^,^5^,^6^. The wide range of trophic interactions and the inherent variability of nutrient fluxes in soil give rise to the formation of microbial consortia that are considered important for maintaining stable ecological interactions within a complex and dynamic soil environment^7^,^8^,^9^. Unlike interactions in well-mixed cultures, the large heterogeneity of diffusion pathways coupled with trophic interdependencies may dictate spatial self-organization for efficient exploitation of physico-chemical niches and for carrying out complex functions such as the biodegradation of compounds that require cooperation of multiple microbial species^8^,^9^,^10^,^11^. Not surprisingly, the spatial context of microbial interactions and the resulting self-assembly that commonly occur in natural systems remain poorly understood due to limitations of traditional pure culture-based microbiology^11^,^12^. In addition, eecological principles conceived for macro-ecosystems (e.g., involving plants and animals) where trophic interactions shape coexistence, biodiversity and ecological function^13^,^14^ may not capture the high dimensional nature of the nuanced and highly localized trophic interdependencies with rapidly changing microbial community composition that give rise to emergence of stable consortia^11^,^12^,^15^. The establishment of a quantitative framework for systematically linking environmental biophysical processes with multispecies microbial interactions in their natural spatial and temporal context is considered essential to advance our understanding of environmental microbiology^4^,^12^,^16^ and serves as the main objective of this study. We report a mechanistic modeling study of cell-level tropic interactions among microbial populations inhabiting hydrated soil surfaces, focusing on how trophic interdependencies are reflected in cell-level interactions and give rise to spatial self-organization of motile microbial communities. The model resolves local nutrient diffusion in space and time that shape cell-level nutrient interception, growth, and interactions among neighboring cells and their local environment. The fragmented and highly dynamic aqueous phase that support microbial community function presents radically different constraints to cell motion and interactions than the more familiar water replete biofilm studies^17^,^18^,^19^,^20^. The study expands on the recent observations and models for spatial pattern formation^21^ by considerations of life and interactions on physically constraining hydrated surfaces, and by considering various trophic interactions that drive formation of stable community patterns^20^. These expand the range of interactions previously studied such as the emergence and dispersion of successful mutants^21^,^22^, or protective segregation of cooperative organisms studied in Hallatschek *et al.*^21^.

A systematic evaluation of processes giving rise to spatially self-organized microbial consortia was based on a mechanistic modeling framework built on previous studies^6^,^23^. The present study combines concepts from microbial biology with physical representation of hydrated soil surfaces enabling mechanistic quantification of microbial nutrient uptake, growth, movement, and interactions with neighboring cells at their local (and dynamic) environments. These are essential ingredients for understanding microbial life in the natural concourse^11^. We first studied the simple case of a consortium consisting of two species (sp1 and sp2) that utilize two obligatory nutrients (*N1* and *N2*) in complementary fashion. We then added complexity with a third species (sp3) that utilizes a by-product (nutrient 3 − *N3*) excreted by the two species as a food source in commensal or mutualistic relations (see Table 1).

## Results and Discussion

**Figure 1:**
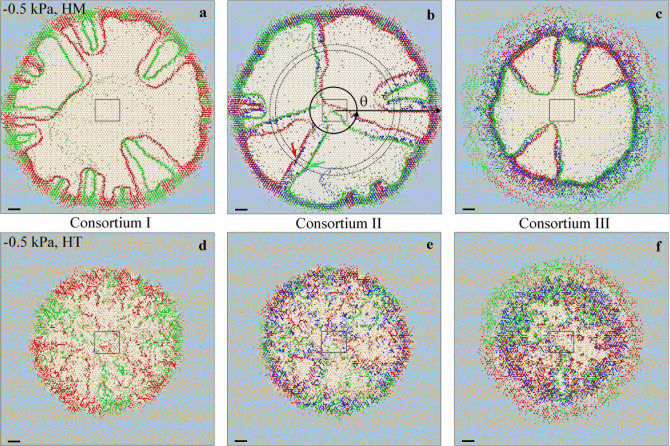
Simulated microbial spatial patterns on rough surfaces of different consortium types. Simulated microbial spatial patterns of consortium I (a, d), consortium II (b, e), and consortium III (c, f) on homogeneous (HM) and heterogeneous (HT) hydrated rough surfaces at −0.5 kPa at 50 h after inoculation. Red, green and blue spots represent individual cells of sp1, sp2 and sp3, respectively. Light blue background marks normalized concentration of *N1* (white area means *N1* was depleted). Squares mark original inoculation positions; a definition of *θ* is for angular distributions of microbial populations and associating nutrient concentrations in Fig. 3, with the dash-circles mark the target region for analyses; red and green arrows mark the persisting population bands of sp1 and sp2. The scale bar is 1 mm.

**Figure 2:**
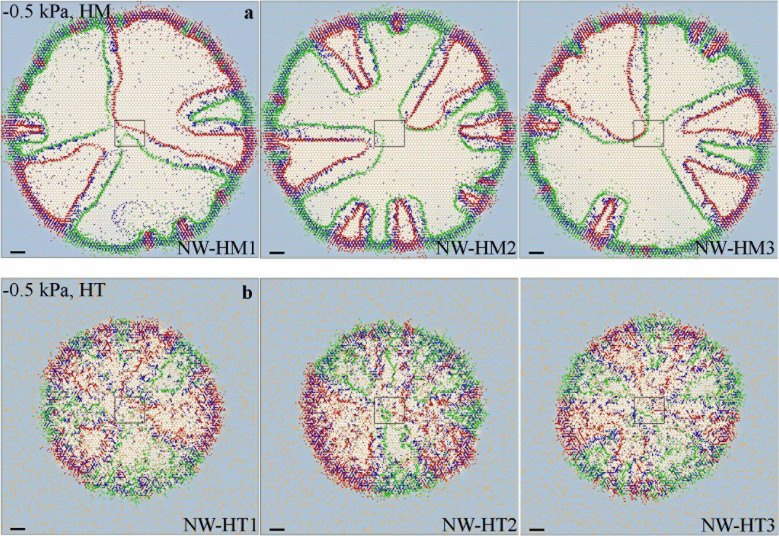
Simulated microbial spatial patterns on homogeneous and heterogeneous rough surfaces. Simulated spatial patterns of microbial consortium II on (a) homogeneous (HM) and (b) heterogeneous (HT) surfaces at −0.5 kPa, at 50 h after inoculation, on three newly generated networks [network-1 (NW-HM1 and NW-HT1), network-2 (NW-HM2 and NW-HT2) and network-3 (NW-HM3 and NW-HT3)]. Red, green and blue spots represent individual cells of sp1, sp2 and sp3, respectively. Light blue background marks normalized concentration of *N1* (white area means *N1* was depleted). Squares mark original inoculation positions. The scale bar is 1 mm.

**Figure 3:**
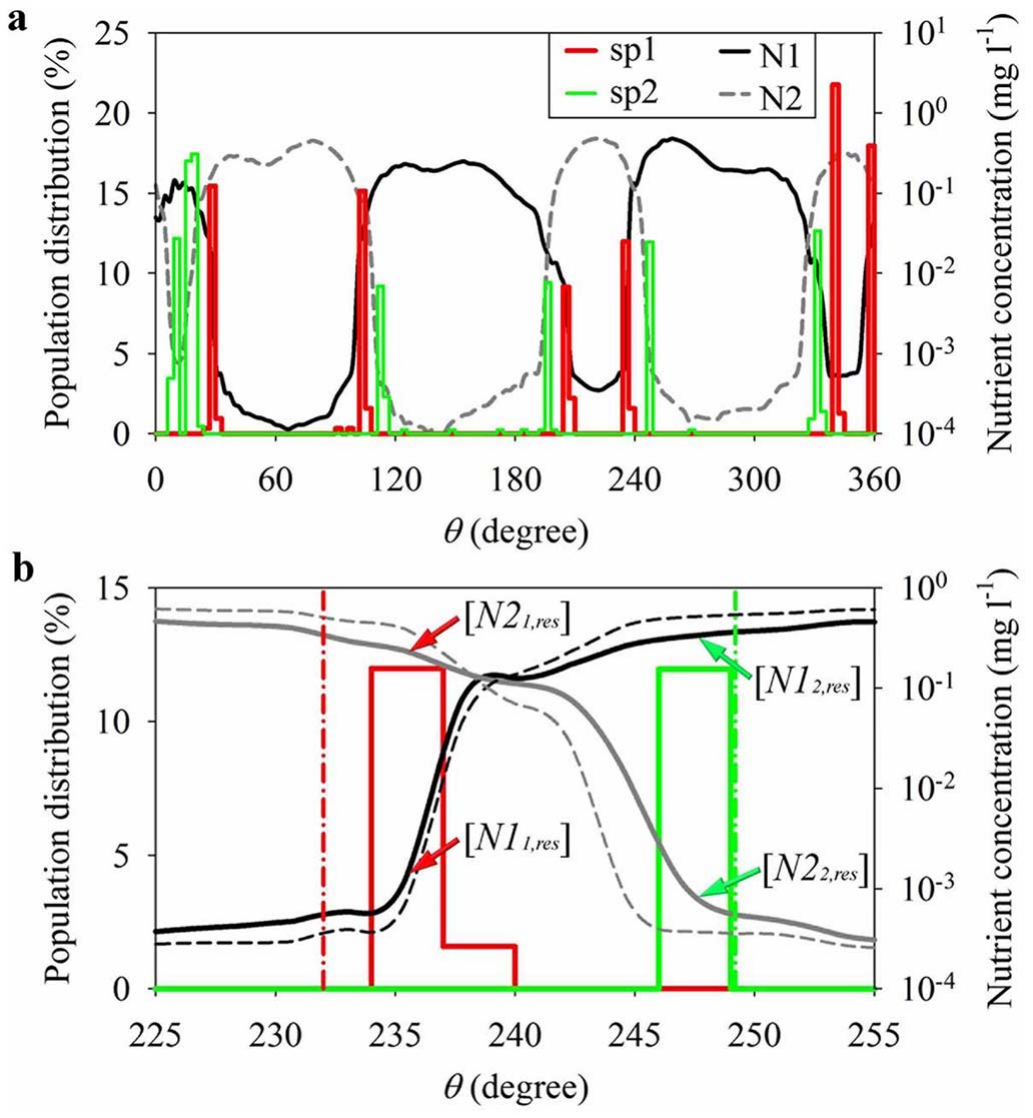
Angular distributions of microbial population fraction and associating nutrient concentrations forming during self-assembly of simulated consortia patterns. (a) Angular distributions of populations sp1 and sp2 and nutrient concentrations *N1* and *N2* at 50 h after inoculation at the region marked by dash-circles (also definition of *θ*) in Fig. 1b; and (b) a zoom-in image of panel (a) with *θ* ranges from 225 to 255 degree (solid black and gray curves mark nutrient concentrations of *N1* and *N2* at 50 h after inoculation, and dash curves for 35 h), and dash-dot lines mark the theoretical positions with the ratio of [*N1_i,res_*] to [*N2_i,res_*] of 0.0015 (0.0062 for simulation, marked by red arrows) for sp1 (red), and 672 (412 for simulation, marked by green arrows) for sp2 (green).

We first considered trophic interactions between two microbial species (sp1 and sp2) with different apparent yields to two obligatory nutrients (*N1* and *N2*) present in the aqueous phase of a homogenous rough surface. The mechanistic modeling of growth, multiplication and chemotactic motility of microbial cells of the two populations spontaneously gave rise to formation of spatially segregated sectors populated by single species, as shown in Figs. 1 and 2. The resulting spatial patterns were remarkably stable irrespective of population growth within a sector as evidenced by the persistent angular distributions of microbial populations (seeing Fig. S1, and Supplementary Movie 1), and by calculated segregation index^27^ values of sp1 and sp2 (for consortium I as an example), which decreased rapidly with elapsed time, and remained stable since 10 hours after inoculation on homogeneous and heterogeneous surfaces (Fig. S2). The segregation and spatial patterns were linked to differences in physiological yields for the two nutrients and the spatial patterns of local concentrations that resulted from preferred nutrient uptake. Interestingly, differences in nutrient utilization resulted in leaky trails of nutrients (see Supplementary Moves 2 and 3) indicative of consumption efficiency commensurate with the prescribed stoichiometry and complementary nutrient utilization by the dominant species in each sector. These nutrient trails, and the presence of both nutrients at sector boundaries, induced the formation of internal microbial population bands (Fig. 3, and Supplementary Movie 2). The populated bands of the two microbial species maintained a relatively stable separation distance that reflected optimal concentration peaks based on the stoichiometric relations of each species and their respective nutrient yields. In other words, each species flourished at the optimal combination of concentrations of the obligatory *N1* and *N2* along opposing gradients of these two nutrients in adjacent sectors. The results depicted in Fig. 3 show agreement between the ratios of local nutrient concentrations of *N1* and *N2* at the sector boundaries due to spontaneous self-assembly, and the theoretical values based on trophic stoichiometry illustrating the strong coupling between physiological traits, trophic dependencies and spatial organization.

Next, we introduced a third species (sp3) into the virtual inoculum where sp3 relies exclusively on a by-product (*N3*) produced by both sp1 and sp2, forming either commensal^25^ or mutualistic^26^,^28^ trophic interactions among the three species (see Table 1). For the commensal scenario, the addition of sp3 did not alter the original spatial self-organization of sp1 and sp2 (in the absence of sp3), and the sp3 population simply followed sp1 and sp2 (Fig. 1). The simulated patterns are reminiscent of spatial self-assembly of microbial consortia observed in other microbial systems such as on moist leaf surfaces^29^ or agar surfaces^30^. A scenario where the increased concentration of the by-product plays an inhibitory role^24^ for the simulated growth of sp1 and sp2 (i.e., a transition into mutualistic interactions) was clearly manifested in the resulting spatial pattern of the stable consortium. A narrow and loosely populated front consisting of sp1 and sp2 only formed ahead of the expanding main population front, and the spatial segregation between trailing internal population bands of sp1 and sp2 has been reduced (Fig. 1). The results illustrate the consequences of mutualistic trophic dynamics in which inhibition at high by-product concentrations depresses growth of sp1 and sp2, “dilutes” population density at the front, and forces a tighter and denser association with sp3 at the perimeter of the consortium. The role of mutualism in promoting soil microbial diversity has been considered previously^28^, and the results here illustrate a spatial manifestation of mutualistic trophic interactions.

The additional constraint generally retarded consortium expansion and resulted in proximal internal population bands of sp1 and sp2 that persisted at a close distance from each other due to the depressed “leaky” concentrations (Supplementary Movie 3). Additionally, to quantify the role of chemotaxis on microbial spatial self-organization, we conducted simulations with random cell motions. The results depicted in Fig. S3 showed complete mixing and limited spatial segregation among trophically interdependent populations for the same conditions listed in Table 1. The results highlight the importance of chemotaxis for the emergence of spatially self-organized microbial consortia.

Surface heterogeneity and patchy nutrient concentrations due to hydration dynamics and complex diffusion pathways are the rules in natural terrestrial systems^1^,^2^,^3^,^6^,^12^. We evaluated effects of surface heterogeneity and hydration status on consortia spatial patterns for similar trophic interactions considered above. The results illustrated emergence of spatially self-organized microbial spatial patterns similar to those forming on homogeneous surfaces (Fig. 1, and Supplementary Movie 1) albeit with suppressed population sizes and constrained dispersion even for mildly wet (−2.0 kPa) and heterogeneous surfaces (Fig. S4). No organized microbial spatial patterns emerged under drier conditions (−5.0 kPa, Fig. S4c) presumably due to suppression of microbial motility, an essential ingredient for growth and self-organization on hydrated surfaces^3^,^6^,^23^. The results suggest a narrow range of hydration conditions (a few kPa of matric potential) that support microbial self-motion in aqueous films^31^ and enable self-assembly of spatially ordered consortia. Equally important, the results indicate that the drivers for self-assembly of microbial populations on heterogeneous surfaces remain dominant despite limitations to motility and fragmentation of nutrient diffusion fields. In other words, trophic interactions and nutrient supply boundary conditions are likely to support microbial consortia, whose spatial pattern optimizes nutrient interceptions even within heterogeneous and patchy nutrient fields as seen, for example, consortium II on a heterogeneous surface (Fig. S5a). We conducted additional tests for spatial self-organization of trophically-interacting microbial populations based on experimentally derived parameters for commensal^25^ (Consortium IV) or mutualistic^26^ (Consortium V) microbial consortia. The modelling results showed well-defined spatial self-arrangement for both commensal and mutualistic microbial consortia (Figs. S5b and S5c) lending support to the generality of the principle of emergence of spatial self-assembly of interacting microbial populations. The results reflect the importance of specific metabolic stoichiometry within multi-nutrients environments that result in trophic-induced niche partitioning^32^,^33^ for promoting stable microbial spatial patterns.

**Figure 4:**
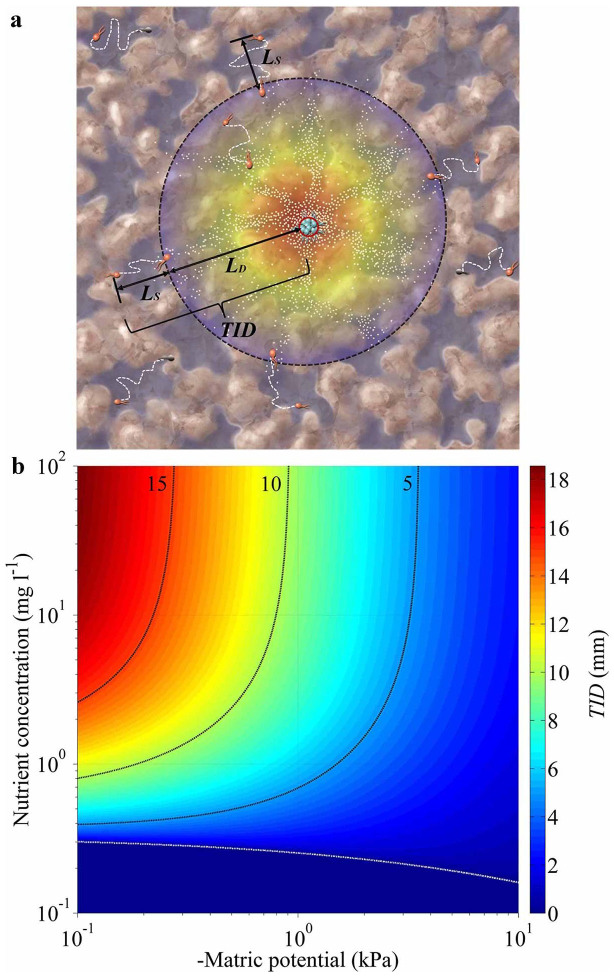
Analytical predictions of microbial *Trophic Interactions Distance (TID)* for the onset of consortium self-assembly. (a) A schematic of the biophysical parameter *TID* and its two components of *L_S_* and *L_D_*, see equations (S10) and (S12), dark dash-circle [or white dash-line in panel (b)] marks threshold concentration of by-product (gray dots with red-yellow-blue colors representing concentration gradient, and red circle marks initial nutrient source) excreted by producer species (light-blue rods) supporting maintenance of consumer species (red rods, white dashed lines marking cell motion trajectories) and triggering trophic interactions and subsequent consortia self-assembly (faded rods mark consumer cells beyond the *TID* range), and dark-blue in the background mark water configuration; and (b) analytically-derived *TID* values at various hydration (expressed as water matric potential value) and nutrient conditions.

The study contributes to the understanding of mechanisms for spatial self-organization of microbial communities^34^, by elucidating the joint roles of a threshold nutrient levels^34^ (in excess of cell maintenance requirements), and of microbial chemotactic motion^35^. Even without resolving the ubiquity of these conditions for microbial pattern formation in the broader ecological context, we can identify a theoretical biophysical characteristic length (maximal separation distance) for the onset of microbial community self-assembly. We derived an analytic prediction of such characteristic length termed the *trophic interactions distance* (*TID*) that defines the maximal initial separation distance between consortium members for the activation of trophic interactions and self-organization (Fig. 4a). The *TID* links hydration-mediated nutrient diffusivity with microbial motility ranges, and thus provides a predictive metric for the onset of consortium self-assembly on rough surfaces. Conditions resulting in large *TID* values (namely rapid cell migration or low threshold concentrations) suggest potential for self-assembly even for low densities or large separation distances among community partners; whereas low *TID* values indicate that consortium members must reside within this limited range to trigger self-assembly (Fig. 4b). This biophysical metric could be useful for design of bioremediation activities including selection of optimal water content, and volumetric densities and strengths of bio-stimulation. Similar estimates may be used for estimating quorum sensing ranges and thresholds^36^,^37^ in soil and other domains.

The growing interest in microbial spatial association as an important ingredient for better understanding of natural microbial ecology has not yet been fully integrated into practical models due to limited experimental information^8^,^9^,^11^. The proposed modeling framework offers an exploratory platform for guiding information gathering and systematic evaluation of trophic dependencies and potential spatial patterns in the context of natural hydrated surfaces that could contribute to anchoring environmental microbiology back in the natural concourse^11^. The results show that cell-level interactions among multispecies with different trophic dependencies induce dynamic and heterogeneous localized nutrient patterns that have been observed in natural systems^2^,^3^,^4^,^11^. Considering the relatively limited temporal window of favorable hydration conditions that support microbial self-organization (for many geographic and climatic regions lasting only several hours a few times per year), the resulting stable spatial patterns could be viewed as rudimentary ecological templates for the more permanent microbial colonies forming on newly inhabited soil surfaces^2^,^3^,^5^,^6^. The results may also offer guidance for acquiring experimental observations to enhance understanding of functioning microbial communities^11^,^12^. The proposed biophysical *TID* for triggering spatial patterns^11^ may have practical applications for the design of artificial microbial consortia in the context of synthetic biology^22^, and for improving efficiency of bioremediation activities in the natural environment^12^,^38^.

## Methods

### Representing hydrated soil surfaces

We developed a spatially-explicit and individual-based model^17^,^23^ to systematically study the spatial and temporal dynamics of multispecies cell-level trophic interactions in the context of the self-assembly of microbial consortia on hydrated surfaces. The simulation domain abstracts natural soil surfaces into a two dimensional network of roughness/capillary elements arranged on a regular lattice^6^,^23^ comprised of 100 × 87 sites spanning a domain with physical size of 17.2 × 17.2 mm. We considered homogeneous roughness networks (HM) consisting of identical roughness elements/channels, and equivalent heterogeneous networks (HT) consisting of heterogeneous roughness elements drawn from a statistical distribution of sizes.

### Modeling microbial growth kinetics and self-motion of individual cells

Microbial growth rate and metabolic reactions for two nutrients limiting growth were described by Monod-type kinetics as^39^, 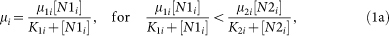
and 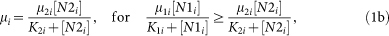
where *μ_i_* (*i* = 1, 2) is effective microbial specific growth rate, *μ_1i_* and *μ_2i_* are the actual specific growth rates, and *K_1i_* and *K_2i_* are half-saturation constants for the first and second nutrients of *N1_i_* and *N2_i_* for species *i*, respectively (see details in Supplementary Information). Diffusion of nutrient within the hydrated roughness network was solved based on Fick's law for the domain updated by microbial nutrient uptake within each roughness element according to the reaction-diffusion model^6^,^23^,^40^.

The self-motion of microbial cells is an important trait that confers advantages for survival in patchy and heterogeneous environments^1^. Self-motion also promotes other biophysical interactions such as self-organization in response to chemotactic gradients^23^,^41^,^42^. Flagellated and other forms of cell motions^41^ on soil surfaces become rapidly restricted with reduction in soil aqueous phase content. These physical restrictions are attributed to enhanced cell-wall viscous drag in thin films, followed by capillary pinning as air-water interfaces interact with microbial cells in unsaturated soil^3^,^23^. The effects of surface hydration state on individual cell motion and on population dispersion rates were expressed by relationship between cell size and effective water film thickness^23^, *d*(*ψ*). For a given matric potential value (*ψ*), the resulting cell velocity (*V*) considering capillary and hydrodynamic limitations is obtained as^6^: *V*(*ψ*) = *V*_0_(*F_M_* − *F_C_*(*d*(*ψ*)) − *F_λ_*(*d*(*ψ*)))/*F_M_* (details see Supplementary Information). For cell motions in response to chemotactic gradients^1^,^42^, we first evaluate the hydration-constrained mean cell velocity, *V*(*ψ*), as a function of local aqueous film thickness. Next, we assign a displacement vector that depends on nutrient (chemo-attractant) gradient by weighing chemotactic and random motility components using complementary weight factors, *ζ* and 1-*ζ*, where *ζ* is the normalized dimensionless nutrient gradient defined as the ratio of local to maximal nutrient gradients, with *ζ* = 0 for entirely random cell motility^6^ (details see Supplementary Information).

### Simulated hydration and heterogeneity scenarios

Microbial population interactions on homogeneous or on heterogeneous hydrated surface roughness networks^6^,^31^ were simulated for different hydration conditions expressed by water matric potential values of −0.5, −2.0 and −5.0 kPa, each with three replicates (we reported −0.5 kPa only for homogeneous scenarios). These hydration conditions mimic soil hydration ranging from very wet to mildly dry surfaces that limit microbial motility in aqueous films. Mixed microbial populations were randomly introduced onto a 2 × 2 mm region at the center of the domain with each species consisting of 100 individual cells (or 30 cells for each inoculation site for the scenario depicted in Fig. S5a). The initial and boundary conditions included uniform initial distributions of nutrients *N1* and *N2* concentrations (2 mg l^−1^) throughout the simulation domain (of physical size 17.2 × 17.2 mm), while maintaining zero nutrient fluxes across the domain boundaries. The exceptions to these conditions were: (i) the scenario presented in Fig. S5a with fixed point nutrient concentrations of *N2* at the region's interior marked by solid circles; and (ii) for the scenarios in Figs. S5b and S5c – consortia IV and V, where only *N1* was initialized throughout the simulation domain, with zero fluxes at the boundaries of the domain.

### Analysis of microbial spatial segregation

Motile microbial cells may relocate within the aqueous network towards positions that improve their nutrient acquisition^18^. We focus on modelling results for consortium II considering the resulting spatial distributions of sp1 and sp2 (Fig. 1b, with population bands of sp1 and sp2 marked by red and green arrows). Interestingly, complementary population bands of the two microbial species form along the boundaries of the occupied sectors. The population bands were separated by relatively constant distance that reflects the nutrient utilization efficiencies of each species and their specific stoichiometric relations to each of the nutrients. Specifically, each species identified an optimal combination of the two obligatory nutrients concentrations (expressed as [*N1_i,res_*] and [*N2_i,res_*]) to satisfy the following condition, 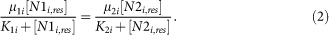
Rearranging equation (2) yields, 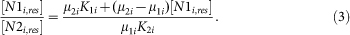
Substituting parameter values for consortium II into equation (3) one obtains, 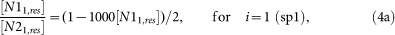
and 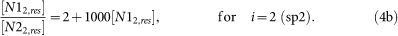
Because the ratio 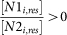 (*i* = 1, 2), the resulting value of [*N*1_1,*res*_] < 0.001[mg l^−1^], and that of [*N*2_2,*res*_] < 0.001[mg l^−1^]. The concentration of residual nutrients within each segregated band (occupied by a single microbial species) can thus be estimated, according to the respective apparent yield for a specific nutrient, as, 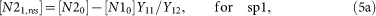
and 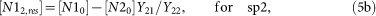
where [*N1_0_*] and [*N2_0_*] are initial nutrient concentrations of *N1* and *N2*, respectively. Substituting the parameter values into equation (5), one obtains [*N2_res_*] = [*N1_res_*] = 0.67 mg l^−1^. Taking into account that [*N*1_1,*res*_] < 0.001[mg l^−1^] and [*N*2_2,*res*_] < 0.001[mg l^−1^], the consumption amount of *N2* for persisting population of sp1 (or *N1* for sp2) is negligible as compared to the values of [*N2_res_*] = [*N1_res_*] = 0.67 mg l^−1^ according to microbial nutrient consumption stoichiometry (see Table 2). Therefore, one may set [*N2_1,res_*] = [*N2_res_*] = 0.67 for sp1, and [*N1_2,res_*] = [*N1_res_*] = 0.67 for sp2; and obtain [*N1_1,res_*] = 0.000997 for sp1, and [*N2_2,res_*] = 0.000997 for sp2 by solving equation (4). Substituting these values into equation (4), one obtains 
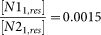
 for sp1, and 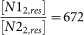 for sp2. These theoretical predictions (based entirely on microbial growth kinetics) were in very good agreement with simulated values associated with spontaneous spatial self-organization that resulted in ratios of 0.0062 for sp1, and 412 for sp2 (see Fig. 3). Considering more general scenarios for environments with low nutrient concentrations, e.g., [*N*1*_i_*] ≪ *K*_1*i*_ and [*N*2*_i_*] ≪ *K*_2*i*_, common in natural environments^3^,^7^,^44^, equation (3) can be simplified as, 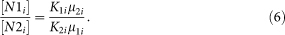
The degree of microbial spatial segregation can be quantified according to a segregation index by Belmonte *et al.*^27^, 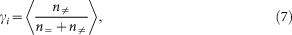
where *n*_≠_ and *n*_ = _ are numbers of neighboring channels dominated by different and same population of species *i*, respectively (with species *i* dominates the target channel), and 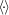 donates an average over the channels that are dominated by species *i*.

### Analytical prediction of critical trophic interactions distance (TID)

The spatial self-organization of microbial consortia emerges through collective interactions among individual cells of consortium members and their local aqueous and nutrient environments. These interactions are shaped by acquisition of essential nutrients and by other environmental stimuli^45^. The spatial separation between the initially unorganized yet trophically interlinked consortium members is critical for the triggering of subsequent spatial self-organization. The key biophysical quantify of interest here, is the critical distance for activation of trophic interactions. We refer this characteristic distance as trophic interactions distance (*TID*), and it defines the maximal initial separation distance between consortium members for activation of trophic interactions calculated as (see details in Supplementary Information), 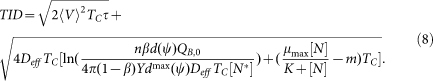
The TID reflects the interplay of hydration-mediated diffusion and motility, and threshold concentrations for setting the conditions for self-assembly and formation of consortium on heterogeneous rough surfaces.

## Supplementary Material

Supplementary Information Movie 1

Supplementary Information Movie 2

Supplementary Information Movie 3

Supplementary Information Supplementary Information

## Figures and Tables

**Table 1:** Schematic presentation of the trophic interactions and consortia

Consortium I	An artificial microbial consortium with species 1 (sp1) and species 2 (sp2) utilize two obligatory nutrients (*N1* and *N2*) with different affinities (marked by different arrows, with solid thick arrows represent large specific growth rates and thinner ones represent smaller growth rates, the same for all schemas).
	
Consortium II	An artificial microbial consortium with both sp1 and sp2 produce (marked by empty arrows, the same for all schemas) an additional nutrient (*N3*) that supports growth of species 3 – sp3 (forming a commensal consortium).
	
Consortium III	An artificial mutualistic microbial consortium, with *N3* toxic to sp1 and sp2. An inhibition form of the Monod equation for microbial growth with presence of by-product S* is expressed as^24^, *K_I_* is inhibition constant, with conditional arrows mark inhibition function of *N3* on sp1 and sp2's growth (the same for all schemas).
	
Consortium IV	Model commensal microbial consortium^25^sp1: *Chlorobiphenyls* → *Chlorobenzoate*sp2: *Chlorobenzoate* →degradation products
	
Consortium V	Model mutualistic microbial consortium^26^sp1: *PEG* + *O*_2_ → *Glyoxylate* + *CO*_2_ + *H*_2_*O* (*Glyoxylate* toxic to sp1)sp2: *Glyoxylate* + *O*_2_ → 2*CO*_2_ + *H*_2_*O*
	

**Table 2:** Physiological parameters for microbial growth, metabolism and nutrient concentrations

		Values				
		sp1		sp2		sp3
		1^st^	2^nd^	1^st^	2^nd^	
Parameters	Units	N_1_	N_2_	N_2_	N_1_	N_3_
*μ_max_*: maximum specific growth rate	hr^−1^	1.2	0.6	1.2	0.6	1.2
*Y_max_*: apparent yield	g dry mass (g nutrient)^−1^	0.44*	0.66	0.44	0.66	0.44
*K_S_*: half-saturation constant	mg l^−1^			1 × 10^−3^†		
*m*: apparent maintenance rate	g nutrient (g dry mass)^−1^ hr^−1^			0.036		
*V_B,0_*: median cell volume	fl			0.4		
*V_B,d_*: cell volume at division	fl			2*V_B,0_*/1.433		
*V_B,min_*: minimal active cell volume	fl			*V_B,d_*/5		
*ρ*: cell density (dry mass)	g l^−1^			290		
*C*: substrate concentration	mg l^−1^			2		
*β*: by-production yield	-			0.5		-
*D*: nutrient diffusion coefficient	mm^2^ hr^−1^			2.4		
*V_0_*: cell velocity	mm hr^−1^			3.6		

*note that negatively correlated growth rate and yield was applied according to Lipson *et al.*^43^.

^†^Small *K_S_* value (as compared to the original value in Kreft *et al.*^17^) was applied to enhance population growth during the limited simulation time; and for analytical calculation in equation (8), it was adjusted to 1 mg l^−1^ based on its original value.
