# Supplementary material for: Trophic interactions induce spatial self-organization of microbial consortia on rough surfaces
Source: Sci Rep. 2014 Oct 24;4:6757. doi: 10.1038/srep06757 (PMC5381366; doi:10.1038/srep06757)
Supplement: Supplementary Information [file srep06757-s4.pdf]

Supplementary Information

Trophic interactions induce spatial self-organization of microbial consortia on rough surfaces

Gang Wang, Dani Or

ETH Zurich, Institute of Terrestrial Ecosystems, Universitaetstrasse 16, 8092 Zurich, Switzerland.

Corresponding author:

Gang Wang (wangethz@gmail.com) and Dani Or (dani.or@env.ethz.ch)

ETH Zurich, Institute of Terrestrial Ecosystems, Universitaetstrasse 16, 8092 Zurich, Switzerland.

Tel: +41 44 633 6013; Fax: +41 44 632 1514

Detailed methods

Representing hydrated soil surfaces

We developed a spatially-explicit and individual-based model[17,23] for systematically studying the spatial and temporal dynamics of multispecies cell-level trophic interactions in the context of the self-assembly of microbial consortia, The simulation domain modeled abstracts natural soil surfaces into a two dimensional network of roughness/capillary elements arranged on a regular lattice[6,23] comprised of 100×87 sites that span a domain with physical size of 17.2×17.2 mm. The aqueous phase within the (capillary) network geometry varies with external conditions (soil matric potential) and gives rise to formation of hydraulically-connected habitats (represented by connected aqueous bonds) that facilitate nutrient diffusion pathways, cell motion and thus the nature of local interactions (nutrient interception, etc.). Previous studies have shown that key transport properties and connectivity of the aqueous phase in the model surface roughness networks mimic macroscopic transport and water holding behavior of soils for different matric potential values (or relative humidity)[23]. To evaluate the role of surface roughness, we considered homogeneous networks (HM, a network consists of identical roughness elements/channels), and equivalent heterogeneous networks (HT, networks consisting of roughness elements drawn from a statistical distribution of sizes).

Modeling microbial growth kinetics

Microbial growth rate and metabolic reactions for conditions where two nutrients limit growth were described by Monod-type kinetics as[39],

$$\mu_i = \frac{\mu_{1i}[N1_i]}{K_{1i} + [N1_i]}, \quad \text{for} \quad \frac{\mu_{1i}[N1_i]}{K_{1i} + [N1_i]} < \frac{\mu_{2i}[N2_i]}{K_{2i} + [N2_i]}, \qquad \text{(S1a)}$$

and

$$\mu_i = \frac{\mu_{2i}[N2_i]}{K_{2i} + [N2_i]}, \quad \text{for} \quad \frac{\mu_{1i}[N1_i]}{K_{1i} + [N1_i]} \geq \frac{\mu_{2i}[N2_i]}{K_{2i} + [N2_i]}, \qquad \text{(S1b)}$$

where μ_i (i = 1, 2) is effective microbial specific growth rate, μ_{1i} and μ_{2i} are the actual specific growth rates, and K_{1i} and K_{2i} are half-saturation constants for the first and second nutrients of $N1_i$ and $N2_i$ for species i, respectively. The apparent nutrient consumption rates of species i for $N1_i$ and $N2_i$ are μ_i / Y_{i1} and μ_i / Y_{i2}, with Y_{i1} and Y_{i2} of apparent yields on $N1_i$ and $N2_i$, respectively. Taking into account microbial cell maintenance and by-product excretion, the effective specific growth rate ($\mu_{eff,i}$) is mediated according to,

$$\mu_{eff,i} = (\mu_i - m_i)(1 - \beta_i), \quad \mu_{eff,i} \equiv 0 \text{ for } \mu_i < m_i, \qquad \text{(S2)}$$

where m_i is microbial specific maintenance rate, β_i is by-production yield defined as a ratio of by-production intake rate to primal nutrient uptake rate, with the by-production intake rate ($r_{N3,i}$) expressed as,

$$r_{N3,i} = (\mu_i - m_i)\beta_i / Y, \quad \text{for} \quad \mu_i \geq m_i, \qquad \text{(S3)}$$

where Y is apparent yield (conversion of nutrients intercepted to biomass). The key physiological parameters were summarized in Table 2 based on the work of Kreft *et al.*[17] and others[25,26]. Diffusion of nutrient within the hydrated roughness network was solved based on Fick's law for the domain updated by microbial nutrient uptake within each roughness element according to the reaction-diffusion model[6,23,40].

Modeling self-motion of individual microbial cells

The self-motion of microbial cells is an important trait that confers advantages for survival in patchy and heterogeneous environments[1]. Self-motion also promotes other biophysical

interactions such as ability to self-organization that wins response to chemotactic gradients[23,41]. Flagellated and other forms of cell motions[42] on soil surfaces become rapidly restricted with reduction in soil aqueous phase content. These restrictions are attributed to enhanced cell-wall viscous drag in thin films, followed by capillary pinning as air-water interfaces interact with microbial cells in unsaturated soil[3,23]. The effects of surface hydration state on individual cell motion and thus on population dispersion rates were expressed by relationship between cell size and effective water film thickness, $d(\psi)$[23]. For a given matric potential value (ψ), the resulting cell velocity (V) considering capillary and hydrodynamic limitations is obtained as: $V(\psi) = V_0(F_M - F_C(d(\psi)) - F_\lambda(d(\psi)))/F_M$, with V_0 is mean cell velocity in bulk water, and F_C and F_λ are the capillary pinning force and the cell-wall viscous drag forces opposing motion driven by the maximum self-propulsion force, and F_M is resistant force in bulk water[6]. Hence, the drying of a soil surface not only impede individual cell motion due to thinning of film thickness, but also results in fragmentation of previously-linked microbial aqueous habitats thereby reducing both ranges of microbial motions and diffusive fluxes[1,3,7,23]. For cell motions in response to chemotactic gradients[1,42], we first evaluate the hydration-constrained mean cell velocity, $V(\psi)$, as a function of local aqueous film thickness. Next, we assign a displacement vector that depends on nutrient (chemo-attractant) gradient by weighing chemotactic and random motility components using complementary weight factors, ζ and 1- ζ, where ζ is the normalized dimensionless nutrient gradient defined as the ratio of local to maximal nutrient gradients, with $\zeta = 0$ for entirely random cell motility[6]. The cell net displacement is expressed as: $\Delta L = (\vec{R}(1-\xi) + \xi)V(\psi)\tau$, with $\vec{R}$ describing direction of random displacement of a cell along (with value of 1) or against (with value of -1) nutrient gradient, and τ is median value of microbial run time.

Simulated hydration and heterogeneity scenarios

Microbial population interactions were simulated on homogeneous or on heterogeneous surface roughness networks[6,31] considering different hydration conditions expressed by the surface matric potential values of -0.5, -2.0 and -5.0 kPa, each with three replicates (we report -0.5 kPa only for homogeneous scenarios). These hydration conditions mimic a range from a very wet surface to a mildly dry surface where self-motion is limited. Mixed microbial populations were randomly introduced on a 2 × 2 mm region from the center of the domain – each species consisting 100 individual cells (or 30 cells for each inoculation site for the scenario in Fig. S5a). The initial and boundary conditions included uniform initial distributions of nutrients *N1* and *N2* concentrations throughout the simulation domain, and maintenance of zero nutrient fluxes across the domain boundaries. The exceptions to these conditions were: (i) the scenario presented in Fig. S5a with point nutrient sources of *N2* at the region's interior marked by solid circles; and (ii) for the scenarios in Figs. S5b and S5c – consortia IV and V, where only *N1* was initialized throughout the simulation domain, with zero fluxes at the boundaries of the domain.

Analysis of microbial spatial segregation

Motile microbial cells may relocate within the aqueous network towards positions that improve their nutrient acquisition[18]. We focus on simulation results from consortium II considering of the spatial distributions of sp1 and sp2 (Fig. 1b, with persisting population bands of sp1 and sp2 marked by red and green arrows). Interestingly, opposing bands of the two microbial species form along the boundaries of the occupied sectors. The population bands were separated by a persistence distance that reflects the nutrient utilization efficiencies of each species and their specific stoichiometric relations to each of the nutrients. Specifically, each species identified an optimal combination of the two obligatory nutrients concentrations (expressed as $[N1_{i,res}]$ and $[N2_{i,res}]$) to satisfy the following condition,

$$\frac{\mu_{1i}\lfloor N1_{i,res}\rfloor}{K_{1i}+\lfloor N1_{i,res}\rfloor}=\frac{\mu_{2i}\lfloor N2_{i,res}\rfloor}{K_{2i}+\lfloor N2_{i,res}\rfloor}. \qquad \text{(S4)}$$

Rearranging equation (S4) yields,

$$\frac{\lfloor N1_{i,res}\rfloor}{\lfloor N2_{i,res}\rfloor}=\frac{\mu_{2i}K_{1i}+(\mu_{2i}-\mu_{1i})\lfloor N1_{i,res}\rfloor}{\mu_{1i}K_{2i}}. \qquad \text{(S5)}$$

Substituting parameter values for consortium II into equation (S5) one obtains,

$$\frac{\lfloor N1_{1,res}\rfloor}{\lfloor N2_{1,res}\rfloor}=(1-1000\lfloor N1_{1,res}\rfloor)/2, \qquad \text{for } i=1 \text{ (sp1)}, \qquad \text{(S6a)}$$

and

$$\frac{\lfloor N1_{2,res}\rfloor}{\lfloor N2_{2,res}\rfloor}=2+1000\lfloor N1_{2,res}\rfloor, \qquad \text{for } i=2 \text{ (sp2)}. \qquad \text{(S6b)}$$

Because the ratio $\frac{\lfloor N1_{i,res}\rfloor}{\lfloor N2_{i,res}\rfloor}>0$ (i=1, 2), the resulting value of $\lfloor N1_{1,res}\rfloor<0.001[\text{mg l}^{-1}]$, and that of $\lfloor N2_{2,res}\rfloor<0.001[\text{mg l}^{-1}]$. The concentration of residual nutrients within each segregated band (occupied by a single microbial species) can thus be estimated, according to the respective apparent yield for a specific nutrient, as,

$$\lfloor N2_{1,res}\rfloor=\lfloor N2_0\rfloor-[N1_0]Y_{11}/Y_{12}, \qquad \text{for sp1}, \qquad \text{(S7a)}$$

and

$$\lfloor N1_{2,res}\rfloor=\lfloor N1_0\rfloor-[N2_0]Y_{21}/Y_{22}, \qquad \text{for sp2}, \qquad \text{(S7b)}$$

where [$N1_0$] and [$N2_0$] are initial nutrient concentrations of *N1* and *N2*, respectively. Substituting the parameter values into equation (S7), one obtains [$N2_{res}$]=[$N1_{res}$]=0.67 mg l^{-1}. Taking into account that $\lfloor N1_{1,res}\rfloor<0.001[\text{mg l}^{-1}]$ and $\lfloor N2_{2,res}\rfloor<0.001[\text{mg l}^{-1}]$, the

consumption amount of $N2$ for persisting population of sp1 (or $N1$ for sp2) is negligible as compared to the values of $[N2_{res}]=[N1_{res}]=0.67$ mg l^{-1} according to microbial nutrient consumption stoichiometry (see Table 2). Therefore, one may set $[N2_{1,res}]=[N2_{res}]=0.67$ for sp1, and $[N1_{2,res}]=[N1_{res}]=0.67$ for sp2; and obtain $[N1_{1,res}]=0.000997$ for sp1, and $[N2_{2,res}]=0.000997$ for sp2 by solving equation (S6). Substituting these values into equation (S6), one obtains $\frac{[N1_{1,res}]}{[N2_{1,res}]}=0.0015$ for sp1, and $\frac{[N1_{2,res}]}{[N2_{2,res}]}=672$ for sp2. These theoretical predictions (based entirely on microbial growth kinetics) were in very good agreement with simulated values associated with spontaneous spatial self-organization that resulted in ratios of 0.0062 for sp1, and 412 for sp2 (see Fig. 1). Considering more general scenarios for environments with low nutrient concentrations, e.g., $[N1_i] << K_{1i}$ and $[N2_i] << K_{2i}$, common in natural environments[3,7,44], equation (S5) can be simplified as,

$$\frac{[N1_i]}{[N2_i]}=\frac{K_{1i}\mu_{2i}}{K_{2i}\mu_{1i}}. \qquad \text{(S8)}$$

The degree of microbial spatial segregation can be quantified according to a segregation index by Belmonte *et al.*[27],

$$\gamma_i=\left\langle \frac{n_{\neq}}{n_{=}+n_{\neq}} \right\rangle, \qquad \text{(S9)}$$

where $n_{\neq}$ and $n_{=}$ are numbers of neighboring channels dominated by different and same population of species *i*, respectively (with species *i* dominates the target channel), and $\langle\cdot\rangle$ donates an average over the channels that are dominated by species *i*.

Analytical prediction of critical Trophic Interactions Distance (TID)

The spatial self-organization of microbial consortia emerges through collective interactions among individual cells of consortium members and their local aqueous and nutrient environments. These interactions are shaped by acquisition of essential nutrients and other environmental stimuli[45]. The spatial separation between the initially unorganized yet trophically interlinked consortium members is critical for the triggering of subsequent spatial self-organization. The analysis identifies the critical distance for activation of trophic interactions as the key biophysical parameter. This critical distance is partially defined by the maximum displacement distance for a cell assuming no nutrients interception (relying entirely on its own inner energy storage, expressed as cell dry biomass) for the physical conditions of the surface[23,46]. An estimate of this distance is given by[47]:

$$L_S = \sqrt{2\langle V \rangle^2 T_C \tau}\,, \qquad \text{(S10)}$$

where $\langle V \rangle$ is microbial median cell velocity on the hydrated rough surface[31], τ is median value of microbial run time[47], and T_C is the survival time of a cell that only utilizes its own stored energy (cell dry biomass) without nutrient supply[48] estimated as,

$$T_C = \frac{1}{m}\ln\left(\frac{Q_{B,0}}{Q_{B,\min}}\right), \qquad \text{(S11)}$$

where $Q_{B,0}$ is the median value of dry biomass of an active cell, and $Q_{B,min}$ is the threshold value of its dry biomass below which a cell turns inactive (a cell switches off its metabolism or simply dies). The other component to this critical distance is determined from the diffusion range (distance) a by-product that a producer species generates. More specifically, it is important to consider a region with concentration values above the threshold required for the maintenance of the consumer species (e.g., sp3). We denote the steady state radius of this maintenance concentration (of the by-product) originating from a producer cell or from a cell

cluster as L_D estimated as[2],

$$L_D = \sqrt{4D_{eff} T_C \ln(Q_N / 4\pi D_{eff} T_C [N^*])}, \qquad \text{(S12)}$$

where D_{eff} is nutrient effective diffusion coefficient on a hydrated rough surface[31], $[N^*]$ is the critical nutrient concentration for consumer cell self-maintenance calculated as, $[N^*] = mK/(\mu_{\max} - m)$, and Q_N is the amount of the point source (dry mass) which we approximated the amount of by-product generated by the producer species as: $Q_N = [\beta/(Y - \beta Y)]Q_{B,T}$, with β is the by-product yield, Y the apparent yield (conversion of nutrient intercepted to biomass), and $Q_{B,T}$ is the total dry biomass of the population of the producer species developed at T_C after inoculation estimated as,

$$Q_{B,T} = [d(\psi)/d^{\max}(\psi)]nQ_{B,0} \exp(\mu_{eff} T_C), \qquad \text{(S13)}$$

where n is the number of cells of the producer colony or cluster modifying or serving as the nutrient source. For illustrative purposes we have selected a value of n=100 for the analysis (similar to the inoculation density in the simulations), noting that the *TID* range is not sensitive to this value across several orders of magnitude of n. The effective specific growth rate of the producer species at nutrient concentration of $[N]$ is expressed as: $\mu_{eff} = \dfrac{\mu_{\max}[N]}{K + [N]} - m$. Note that $Q_{B,T}$ was adjusted according to the total available initial nutrient mass which (for uniform concentration) is proportional to the effective water film thickness of the rough surface[6,23], $d(\psi)$. The parameter $d^{max}(\psi)$ is the maximum film thickness under wet surface condition, considered in this study as thickness under -0.5 kPa of water matric potential. Equations (S11) and (S13) enable the estimation of the *trophic interactions distance* (*TID*) defined as the maximal initial separation distance between consortium members for activation of trophic interactions calculated as (note that for $L_D \leq 0$, $TID \equiv 0$),

$$TID = \sqrt{2\langle V\rangle^2 T_C \tau} + \sqrt{4D_{eff}T_C[\ln(\frac{n\beta d(\psi)Q_{B,0}}{4\pi(1-\beta)Yd^{\max}(\psi)D_{eff}T_C[N^*]}) + (\frac{\mu_{\max}[N]}{K+[N]} - m)T_C]} . \quad \text{(S14)}$$

The *TID* reflects the interplay of hydration-mediated diffusion and motility, and threshold concentrations for setting the conditions for self-assembly and formation of consortia on heterogeneous rough surfaces.

Supplementary Figures S1-S5

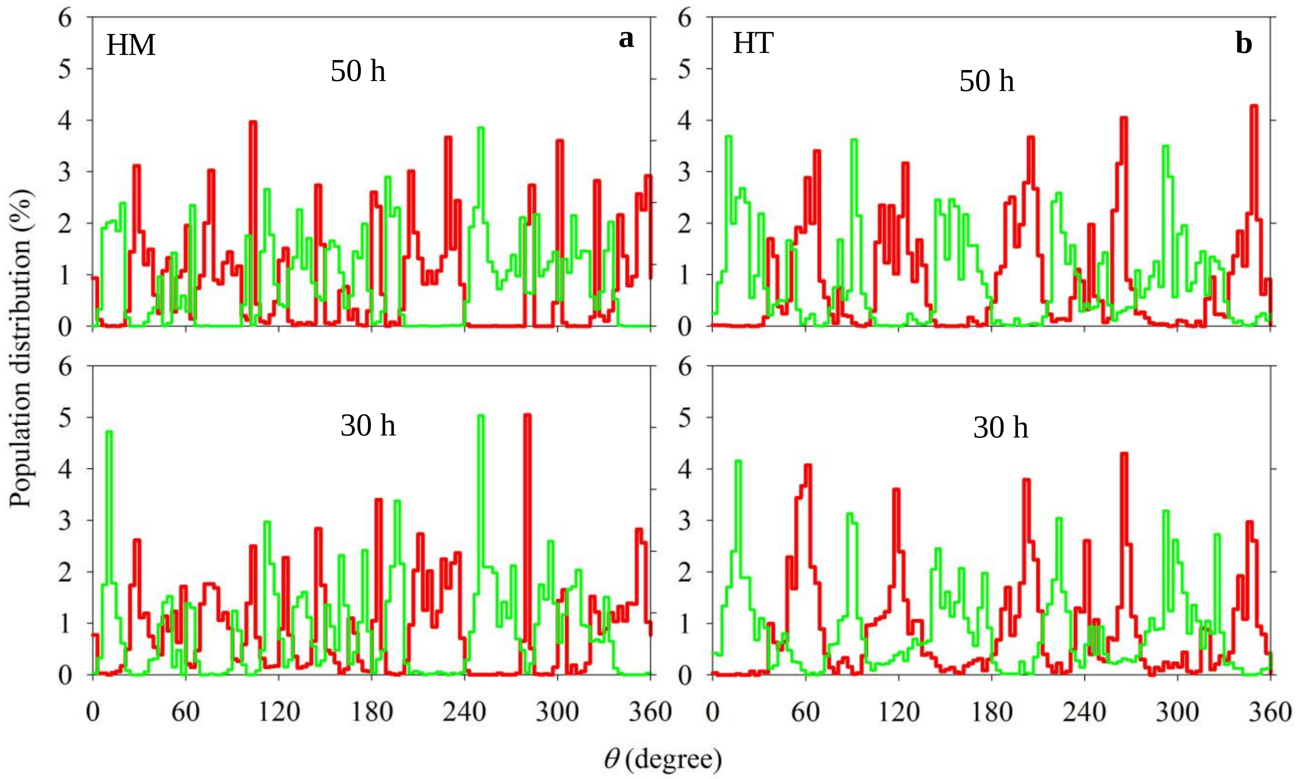

Fig. S1. The angular distributions of microbial populations sp1 and sp2 of a simulated consortium pattern at -0.5 kPa. Simulated angular population distributions (including all the cells at different radii) of sp1 (red) and sp2 (green) of consortium II on (**a**) homogeneous (HM); and (**b**) heterogeneous (HT) hydrated surfaces, at -0.5 kPa at 30 (bottom) and 50 (top) hours after inoculation.

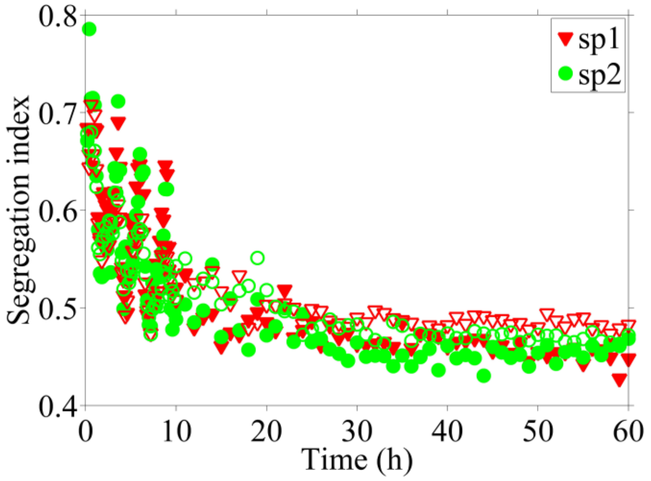

Fig. S2. The evolution of microbial segregation index values over elapsed time on homogeneous and heterogeneous rough surfaces at -0.5 kPa. Evolution of microbial segregation index [equation (S9)] values of sp1 and sp2 of consortium I during the elapsed time since inoculation on homogeneous (close symbols) and heterogeneous (open symbols) rough surfaces at -0.5 kPa.

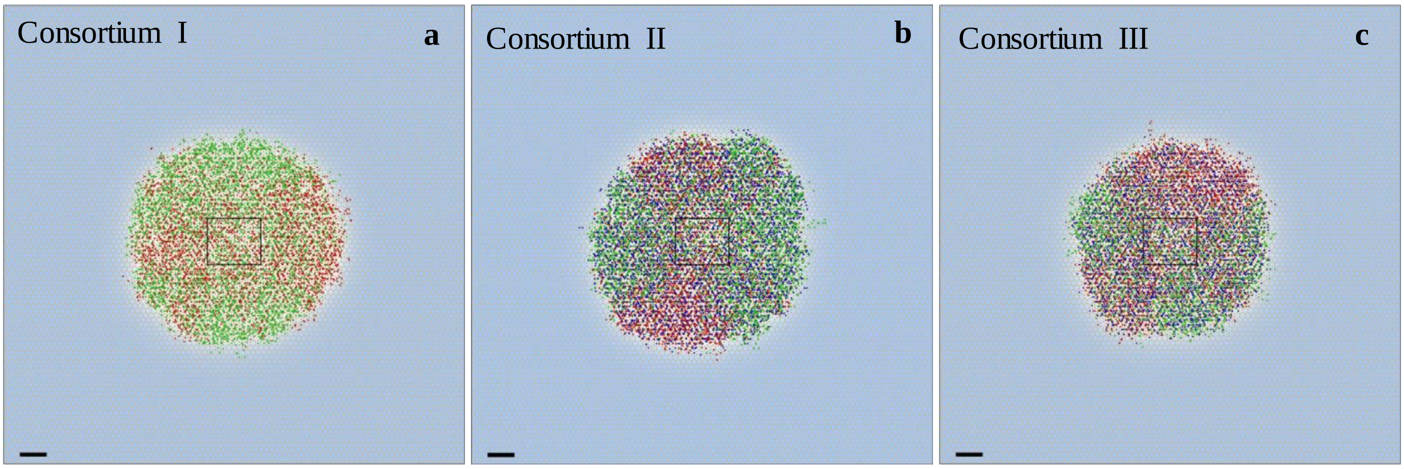

Fig. S3. Simulated microbial spatial patterns of different consortium types due to random cell motion. Simulated spatial patterns of non-chemotactic microbial consortia: (**a**) consortium I; (**b**) consortium II; and (**c**) consortium III on homogeneous (HM) hydrated surfaces at -0.5 kPa at 50 h after inoculation due to random cell motion – in the absence of chemotaxis. Red, green and blue spots represent individual cells of sp1, sp2 and sp3, respectively. Light blue marks normalized concentration of *N1*. Squares mark original inoculation positions. The scale bar is 1 mm.

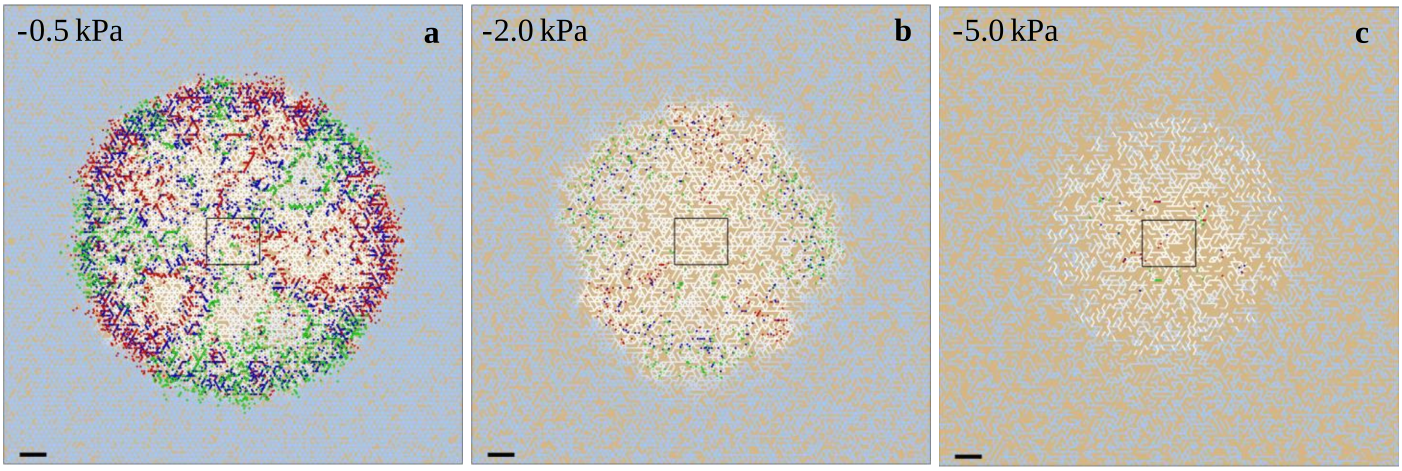

Fig. S4. Simulated microbial spatial patterns on heterogeneous rough surfaces at matric potential values of -0.5, -2.0 and -5.0 kPa. Simulated spatial patterns of microbial consortium II on heterogeneous surfaces at (**a**) -0.5 kPa; (**b**) -2.0 kPa; and (**c**) -5.0 kPa, at 50 h after inoculation. Red, green and blue spots represent individual cells of sp1, sp2 and sp3, respectively. Light blue background marks normalized concentration of *N1* (white area means *N1* was depleted). Squares mark original inoculation positions. The scale bar is 1 mm.

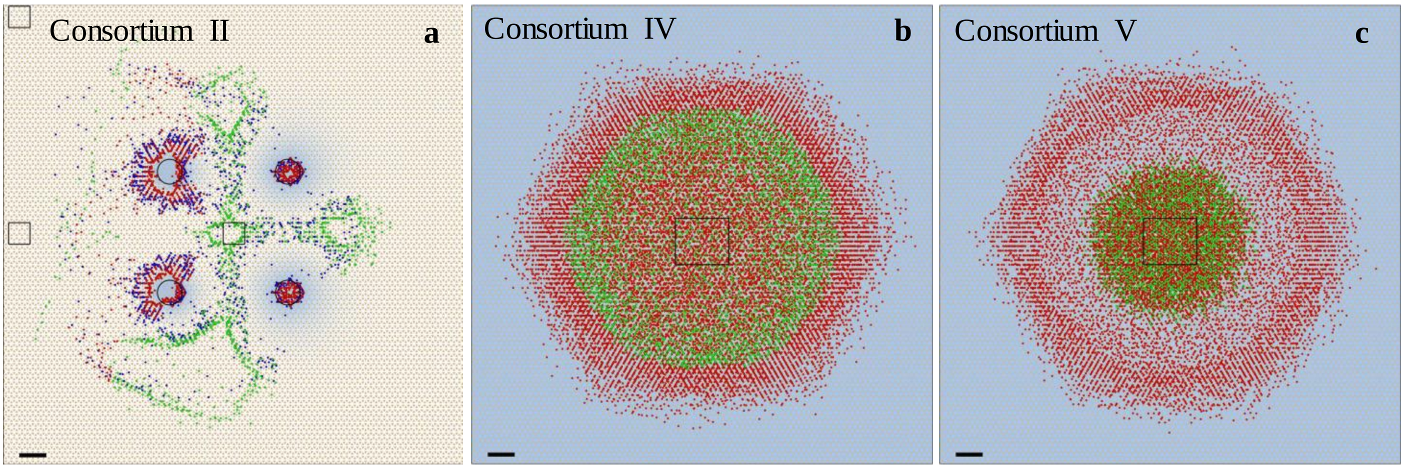

Fig. S5. Simulated microbial spatial patterns on rough surfaces of different consortium types. Simulated spatial patterns of (**a**) consortium II, with spatially structured nutrient fields; and (**b**) consortium IV and (**c**) consortium V that based on real consortia literature data[25,26] with a range of cell motility values, on homogeneous (HM) rough surfaces at -0.5 kPa at 50 h after inoculation. Red, green and blue spots represent individual cells of sp1, sp2 and sp3, respectively. Light blue marks normalized concentration of *N1* (or *N2* in left pattern with cycles marking point nutrient sources of *N2*). Squares mark original inoculation positions. The scale bar is 1 mm.

Movies' Legends

Movie 1. Time-lapse video of spatial self-organization and population growth kinetics of simulated microbial consortia on hydrated surfaces. Simulated spatial self-organization (top) and population growth kinetics (bottom) of trophically interlinked microbial populations of consortium II (Table 1), on homogeneous (HM, left) and heterogeneous (HT, right) rough surfaces at -0.5 kPa. Red, green and blue spots (curves) represent individual cells (populations) of sp1, sp2 and sp3, respectively. Light blue marks normalized concentration of *N1* (white area means *N1* was depleted). The scale bar is 1 mm.

Movie 2. Time-lapse video of angular distributions of microbial populations and associating nutrient concentrations of simulated consortium II. Time-lapse video of angular distributions of microbial populations sp1 (red curve) and sp2 (green curve) and associating nutrient concentrations of *N1* (solid-black curve) and *N2* (dash-gray curve) of simulated consortium II at the region marked by dash-circles (Fig. 1b), with the definition of θ in the same figure.

Movie 3. Time-lapse video of angular distributions of microbial populations and associating nutrient concentrations of simulated consortium III. Time-lapse video of angular distributions of microbial populations sp1 (red curve) and sp2 (green curve) and associating nutrient concentrations of *N1* (solid-black curve) and *N2* (dash-gray curve) of simulated consortium III (Table 1) at the same region of Movie 2.

References

46. Wang, G. & Or, D. Aqueous films limit bacterial cell motility and colony expansion on partially saturated rough surfaces. *Environ Microbiol* **12**, 1363-1373 (2010).

47. Berg, H.C. *Random walks in biology*. Expanded edition. Princeton Univ. Press: Princeton (1993).

48. Russell, J. B. & Cook, G. M. Energetics of bacterial growth: balance of anabolic and catabolic reactions. *Microbiol Rev* **59**, 48-62 (1995).
